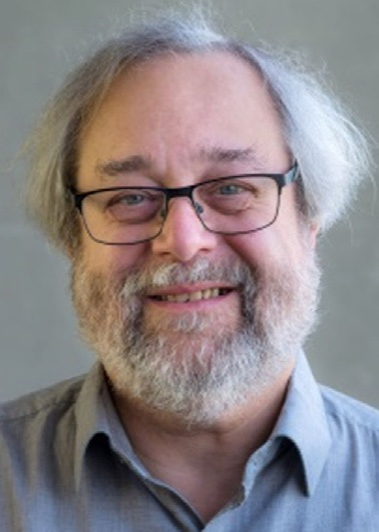# Synthetic Approaches to Triarylboranes from 1885 to 2020

**DOI:** 10.1002/chem.202005302

**Published:** 2021-03-01

**Authors:** Sarina M. Berger, Matthias Ferger, Todd B. Marder

**Affiliations:** ^1^ Institut für Anorganische Chemie and Institute for Sustainable Chemistry, & Catalysis with Boron Julius-Maximilians-Universität Würzburg Am Hubland 97074 Würzburg Germany

**Keywords:** arylmetalate, boranes, chromophore, Lewis acid, synthetic methods

## Abstract

In recent years, research in the fields of optoelectronics, anion sensors and bioimaging agents have been greatly influenced by novel compounds containing triarylborane motifs. Such compounds possess an empty p‐orbital at boron which results in useful optical and electronic properties. Such a diversity of applications was not expected when the first triarylborane was reported in 1885. Synthetic approaches to triarylboranes underwent various changes over the following century, some of which are still used in the present day, such as the generally applicable routes developed by Krause et al. in 1922, or by Grisdale et al. in 1972 at Eastman Kodak. Some other developments were not pursued further after their initial reports, such as the synthesis of two triarylboranes bearing three different aromatic groups by Mikhailov et al. in 1958. This review summarizes the development of synthetic approaches to triarylboranes from their first report nearly 135 years ago to the present.

## Graphical abstract

**This review presents a history** of the methods used to synthesize triarylboranes, which are an interesting class of electron‐poor compounds with a wide range of useful properties and applications as Lewis acids, frustrated Lewis pair (FLP) catalysts, electron transporters, luminescent chromophores for OLEDs, anion sensors, and bioimaging agents, among others.

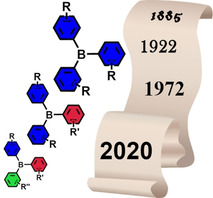

## Introduction

Within the last few decades, compounds containing three‐coordinate boron motifs have found increasing applicability in various fields including optoelectronics,[^1^, ^2^, ^3^] selective sensors for anions[^4^, ^5^, ^6^] or small molecules,[^7^, ^8^] and bioimaging agents[^9^, ^10^, ^11^, ^12^, ^13^, ^14^, ^15^] due to the empty p‐orbital at the boron center. Whereas numerous compounds and their potential applications have been reviewed by several groups,[^16^, ^17^, ^18^, ^19^, ^20^, ^21^, ^22^, ^23^] synthetic methodology for the preparation of triarylboranes has been reviewed only rarely. In 1956, a summary by Lappert et al. gave a very general overview of the syntheses of many different types of organoboron compounds.^[24]^ Very recently, Melen and co‐workers summarized synthetic pathways to halogenated triarylboranes as well as their use in catalysis and frustrated Lewis pair (FLP) chemistry.^[25]^ This review presents developments in the synthesis of triarylboranes since their first report in 1885.^[26]^

In theory, it is possible to synthesize 3‐fold symmetric triarylboranes bearing one type of aromatic system (BAr_3_) as well as those containing two or three different aromatic systems (BAr_2_Ar’ or BArAr'Ar’’). Furthermore, their synthesis should be possible from all known boron trihalides. Recently, the synthesis of triarylboranes from potassium aryltrifluoroborates[^27^, ^28^, ^29^, ^30^, ^31^] and boronic esters^[32]^ was reported. We do not discuss dibenzoboroles^[33]^ or boron‐containing polyaromatic hydrocarbons (B‐PAHs)[^34^, ^35^, ^36^] in our review as both topics have been reviewed recently.

This review is divided into sections based on symmetrically (BAr_3_, BAr_2_Ar’) and unsymmetrically substituted (BArAr'Ar’’) triarylboranes depending on the starting material used as the boron source. In this context, the term unsymmetrically substituted triarylboranes means that the boron center is bound to three different aromatic systems. Symmetrically substituted triarylboranes bear one or two different types of aromatic groups as indicated in parentheses (vide infra) and, thus, have either (exact or approximate) 3‐fold or 2‐fold symmetry, respectively.

## 1 Synthesis of Symmetrically Substituted Triarylboranes

### 1.1 Boron trichloride, tribromide and boronic esters as boron sources

**Scheme 1:**
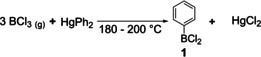
Synthesis of dichlorophenylborane **1** from BCl_3_.^[37]^

In 1880, Michaelis and co‐workers began to investigate arylboranes to determine the valency of boron which was, at the time, debated to be three or five.^[37]^ They reacted gaseous BCl_3_ with diphenylmercury at elevated temperatures in a sealed tube and observed the formation of dichlorophenylborane **1** and HgCl_2_ (Scheme 1). Compound **1** was isolated via distillation and was characterized by elemental analysis and conclusive follow‐up chemistry.

After addition of different aqueous solutions, they obtained phenylboronic acid, the respective ethyl ester and the sodium, calcium and silver salts of the acid, as well as their *p*‐tolyl analogues.^[38]^ On an interesting side note, phenylboronic acid, as well as its sodium salt, were also investigated for their antiseptic behavior, and were consumed by humans on a gram scale without causing any considerable complaints.^[38]^ In 2015,^[39]^ a series of boronic acids and esters were tested using the Ames assay,[^40^, ^41^] and most of them were found to be mutagenic. Thus, this class of compounds should be treated with appropriate care and due testing should be performed prior to use in humans, although several boronic acids or related compounds have been approved for use as drugs.^[39]^

**Scheme 2:**
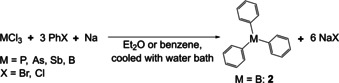
Synthesis of triphenyl derivatives of main group elements.[^26^, ^42^, ^43^]

In a different approach, Michaelis and co‐workers developed a procedure to generate triphenyl derivatives of various main group elements (M), namely phosphorus, arsenic, antimony, and boron.[^42^, ^43^] The respective MCl_3_ compound was reacted with a phenylhalide and elemental sodium at low temperature to generate the corresponding triphenyl compound according to Scheme 2.

In 1885, Michaelis and co‐workers mentioned that, via this general route, a small amount of triphenylborane **2** was obtained, but it was not discussed further.^[26]^ To the best of our knowledge, this was the first literature report of the synthesis of a triarylborane. Four years later, the synthesis of **2** was improved by reacting dichlorophenylborane **1** with chlorobenzene and sodium.^[44]^ This time, compound **2** was characterized by elemental analysis and the appearance of a green flame characteristic of boron^[45]^ when burning the compound.

**Scheme 3:**
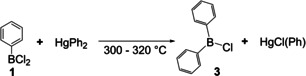
Synthesis of chlorodiphenylborane **3** under harsh conditions.^[47]^

In 1889, Gattermann and co‐workers reported a convenient method for the synthesis of BCl_3_.^[46]^ Thus, access to the starting material was facilitated. Therefore, Michaelis et al. synthesized more dichloroarylboranes and their respective boronic acids, namely the *o*‐tolyl, α‐naphthyl, β‐naphthyl, *p*‐methoxyphenyl, *o*‐methoxyphenyl and *p*‐ethoxyphenyl derivatives.^[47]^ For the latter three compounds, the reaction proceeded smoothly at room temperature. Furthermore, chlorodiphenylborane **3** and its borinic acid derivative were reported and characterized. Compound **3** was formed by reacting dichlorophenylborane with diphenylmercury at ca. 300 °C in a sealed tube (Scheme 3). It was noted that triphenylborane **2** was not obtained this way.

**Scheme 4:**
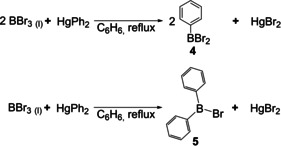
Synthesis of mono‐ and diarylboranes, starting from BBr_3_.^[48]^

In 1901, Michaelis and co‐workers reported an improved method for the synthesis of mono‐ and diarylboranes in which they replaced gaseous BCl_3_ with liquid and, thus, easier to handle BBr_3_ as the boron source.^[48]^ For this purpose, they developed a convenient and large‐scale synthesis of BBr_3_ from elemental boron and bromine. BBr_3_ was then reacted with diphenylmercury in dry benzene. The reaction was performed in a flask with a reflux condenser at 80 °C. Depending on the stoichiometry, dibromophenylborane **4** and bromodiphenylborane **5** were synthesized and isolated via distillation (Scheme 4). Some derivatives, namely dibromo‐*p*‐tolylborane, dibromo‐2,4‐dimethylphenylborane, and dibromo‐2,4,5‐trimethylphenylborane were synthesized and characterized, accordingly. The respective boronic and borinic acids were obtained and characterized after hydrolysis. Again, it was mentioned that, despite extensive studies in this direction, triphenylborane could not be isolated. They assumed that triphenylborane had been formed, but complete separation from diphenyl impurities could not be achieved.

**Scheme 5:**
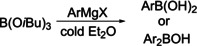
Synthesis of boronic acids and borinic acids, starting from *iso*‐butylborate ester.^[52]^

Another difficulty of arylborane syntheses were the often laborious and multistep syntheses of the required diarylmercury compounds. With the discovery of the Grignard reagent in 1900, a powerful tool for the transfer of aryl groups became available.^[49]^ The first to utilize this in arylborane chemistry, were Khotinsky and Melamed.^[50]^ They treated various alkylborate esters with an aryl Grignard reagent in a cold Et_2_O solution. The best results were obtained for the *iso‐*butylborate ester. Furthermore, Khotinsky and Melamed characterized the phenylboronic *iso‐*butyl ester and the *m*‐tolylboronic *iso*‐butyl ester, as well as the respective boronic acids after saponification. In an attempt to attach two arenes to the boron using Grignard reagents, Strecker reacted an excess of phenyl magnesium bromide with BCl_3_, but obtained only phenylboronic acid after aqueous work up.^[51]^ A more extensive study of the reactions of aryl Grignard reagents with the *iso*‐butylborate ester was carried out by König and Scharrnbeck in 1915. The results were reported in 1930.^[52]^ They characterized several novel arylboronic acids and diarylborinic acids which were synthesized according to Scheme 5 and isolated after aqueous work‐up indicating that the organometallic reagent used was too unreactive to form the corresponding triarylborane. More than 70 years later, it was demonstrated by several groups that triarylboranes can also be synthesized using boronic esters as starting materials and more reactive organometallic reagents (vide infra).[^39^, ^53^, ^54^, ^55^, ^56^, ^57^, ^58^]

### 1.2 Boron trifluoride as the boron source

**Scheme 6:**
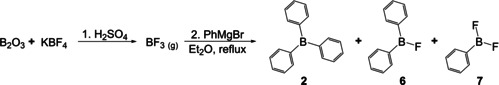
Synthesis of the first reported triarylborane **2** according to Krause et al.^[61]^

Despite reports of the synthesis of triphenylborane,[^26^, ^44^] reproducible synthetic access was only available for mono‐ and diarylboranes in the beginning of the 20th century. This changed with the studies of Krause, who made use of the synthesis of gaseous boron trifluoride (BF_3_) from boric anhydride (B_2_O_3_), sulfuric acid (H_2_SO_4_) and potassium tetrafluoroborate (KBF_4_), reported by Schiff et al.^[59]^ In 1921, Krause and co‐workers used gaseous BF_3_ in combination with Grignard reagents to yield trialkylboranes as well as alkylboronic acids.^[60]^ Subsequently, Krause et al. applied this method for the synthesis of triphenylborane **2** (Scheme 6).^[61]^

**Scheme 7:**
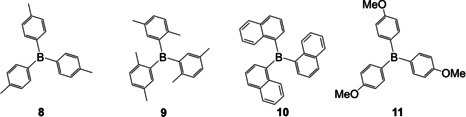
Structures of compounds **8**–**11**.[^64^, ^65^, ^66^]

They isolated BPh_3_ **2** by distillation of the crude reaction mixture in ca. 50 % yield. The product crystallized easily, but it was also mentioned that **2** decomposes in air. Furthermore, Krause and co‐workers observed the formation of phenyldifluoroborane **6** as well as diphenylfluoroborane **7**, but isolation of these two compounds was not possible by distillation. This indicates that the reactivity of the Grignard reagent is insufficient to generate only BPh_3_, as byproducts **6** and **7** were observed. However, with BPh_3_ **2** in hand, the group investigated its reactivity with neat sodium^[62]^ and the other alkali metals potassium, lithium, rubidium and cesium.^[63]^ Krause and co‐workers observed the formation of intensely colored solutions as well as the formation of, mostly, yellow crystals. Both solutions and solids were reported to be highly air sensitive, as the solutions turned colorless when exposed to air. The colorless solution was converted into the colored solution again if neat metal was still present in the solution. After Krause and co‐workers had isolated the reaction product of BPh_3_ **2** with neat sodium,^[63]^ they titrated the reaction product under a nitrogen atmosphere with elemental iodine which regenerated BPh_3_ and sodium iodide. In the same study, the synthesis of tri‐*p*‐tolylborane **8** was mentioned. Its final synthesis and full characterization were reported two years later.^[64]^ Again, the reactivity of **8** with sodium and potassium was investigated as well as its reaction with nitrogenous bases such as ammonia, pyridine, and piperidine. The reaction of **8** with neat sodium was described to be the same as for **2**. During the reactions of **8** with nitrogenous bases, the group observed a temperature increase of the reaction mixture as well as the formation of crystalline and more air‐stable products which were assigned to be addition products of the nitrogenous bases with **8** (Scheme 7). This assumption was confirmed by elemental analysis of the reaction products. In 1930, Krause and co‐workers also reported the synthesis of tri‐*p*‐xylylborane **9** and tri‐α‐naphthylborane **10**, which were investigated similarly to the previous compounds **2** and **8**.^[65]^ For the isolation of **9** and **10**, the work up was slightly modified. Thus, to quench the remaining Grignard reagent, water was added and the resulting crude mixture was distilled with exclusion of air, as none of the previously synthesized triarylboranes are stable to air.

Nevertheless, what they refer to as an “oxidation process” of compound **10** starts only after two weeks in air. Furthermore, Krause et al. reported that solutions of **10** in benzene, chloroform, tetrachloromethane, carbon disulfide, and diethyl ether show a light blueish fluorescence that was more clearly visible with a quartz lamp. They did not provide any further information regarding what kind of lamp or which wavelength they used for excitation. Furthermore, the observed fluorescence was not investigated in detail. In 1931, the same group reported another triarylborane, namely tri‐*p*‐anisylborane **11**, which was found to be as air‐sensitive as triphenylborane **2**.^[66]^ In addition, Krause et al. had to change their work up once again, as they could not isolate **11** in pure form from the crude reaction mixture. Therefore, they reacted a crude mixture of **11** with gaseous ammonia to form the corresponding tetra‐coordinate Lewis acid‐base adduct which was then purified and subsequently reacted with sulfuric acid with exclusion of air to yield compound **11**. Similarly, 20 years later, the same group described the formation of BPh_3_ upon heating different tetraarylborate salts to at least 200 °C.^[67]^ Tetraarylborates are used on rare occasions to this day as valuable, alternative precursors to triarylboranes.[^68^, ^69^, ^70^]

Based on this work, Brown et al. re‐synthesized tri‐α‐naphthylborane **10** as a reference Lewis acid to estimate the Lewis base strength of primary, secondary, and tertiary amines,^[71]^ having slightly modified the synthesis of the triarylborane. To make the synthesis safer, Brown and co‐workers used boron trifluoride etherate (BF_3_⋅OEt_2_) instead of gaseous boron trifluoride. Furthermore, they found that the triarylborane they synthesized was stable to air for more than one year. As this finding was in contrast with the reports of Krause et al.,^[65]^ Brown et al. had a closer look into the geometry of the compound. They assigned the discrepancy between their and the earlier results to the existence of two possible rotational conformers, i.e., steric hindrance resulted in restricted rotation around the B−C bonds.

**Scheme 8:**
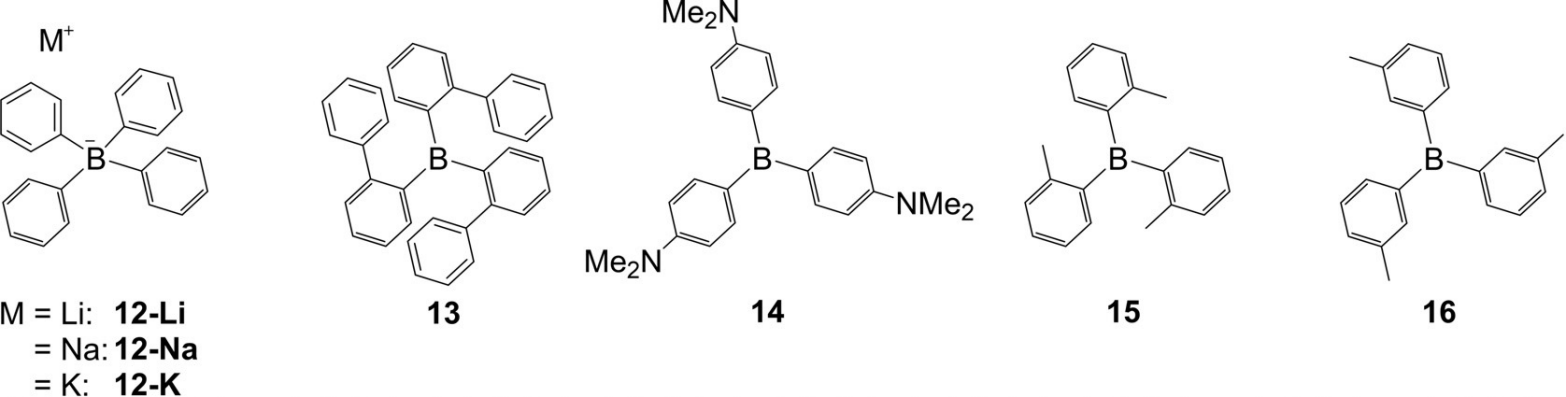
Structures of compounds **12**–**16**.[^73^, ^74^]

In 1947, Wittig et al. investigated the possible application of triphenylborane **2** as a catalyst for the lithiation of hydrocarbons.^[72]^ Instead of successful catalysis of the reaction, they found the formation of a stable complex which was later identified as lithium tetraphenylborate **12‐Li**.^[73]^ Further investigations of such compounds, especially the reaction of sodium tetraphenylborate **12‐Na** with various mono‐cationic elements in aqueous solution, led to the discovery of an almost insoluble complex **12‐K** formed after addition of potassium salts. Later on, compound **12‐Na** became commercially available as Kalignost^®^ for the quantitative analysis of potassium in aqueous solution (Scheme 8).^[74]^

Wittig and co‐workers found that triarylboranes such as **2** can also be synthesized from the corresponding, more reactive lithiated species instead of the Grignard reagent.^[73]^ As long as it was possible to synthesize the desired compounds from Grignard reagents and BF_3_ etherate, they did so. However, for tri(*o*‐diphenylyl)‐ **13** and tri(4‐(*N*,*N*‐dimethylamino)phenyl)borane **14**, Wittig et al. used the corresponding aryllithium reagent.^[74]^ Nevertheless, the synthesis of **14** was still challenging, as the amine formed complexes with excess BF_3_. Furthermore, this group reported a yellowish fluorescence from **14** in the solid state as well as a blue fluorescence in acetone upon irradiation with UV light. Compounds **15** and **16** were described as having a yellowish‐white fluorescence upon UV‐irradiation. None of these observations were further explained or investigated by Wittig et al. Very recently, Marder and co‐workers reported that a sample of pure **16** showed only blue fluorescence, with no phosphorescence being observed at room temperature.^[75]^

In 1956, Lappert summarized the preparation, chemical and physical properties, reactivities, etc. of almost all organoboranes that had been synthesized up to that date.^[24]^ In this summary, several methods to synthesize monohaloboranes as well as unsymmetrically substituted diaryl borinic esters were described. However, almost none of these syntheses were utilized for the formation of triarylboranes, especially not for the formation of boranes bearing three different aromatic systems.

**Scheme 9:**
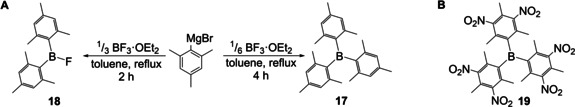
A) Reaction sequences for the synthesis of compounds **17** and **18**.^[76]^ B) Structure of compound **19**.^[77]^

One year later, Brown et al. reported the synthesis of the sterically demanding trimesitylborane **17** from the corresponding Grignard reagent and BF_3_ etherate.^[76]^ The group heated the reagents in toluene under reflux for 4 h which they described as forcing conditions. If the reaction was stopped after 2 h, only fluorodimesitylborane **18** was isolated showing once again that the formation of triarylboranes from Grignard reagents is possible, but requires heat to achieve completion due to the lower reactivity of arylmagnesium reagents compared to, e.g., aryllithium reagents (Scheme 9). Furthermore, Brown and co‐workers examined the reactivity of **17** with amines as well as its decomposition with water and oxygen. It was found that **17** was less reactive than tri‐α‐naphthyl‐ **10** or triphenylborane **2** due to its greater steric hindrance.

Subsequently, the syntheses of compounds **17** and **18** were further improved by Hawkins et al.^[77]^ who changed the solvent for the formation of the Grignard reagent from diethyl ether to THF according to a general procedure reported by Ramsden et al.^[78]^ This change resulted in a shorter reaction time for the formation, as well as an increased yield, of the Grignard reagent. In addition, this led to the isolation of fluorodimesitylborane **18** in 96 % yield. Due to its steric hinderance, the reaction of excess mesityl Grignard reagent with BF_3_ etherate at 55 °C stops at the fluorodimesitylborane stage as long as the reaction time is shorter than 2 h. Furthermore, Hawkins and co‐workers were able to nitrate **17** to yield compound **19**.

In 1967, at Eastman Kodak, Grisdale and co‐workers began to investigate the photophysical reactions of tetraarylborates and triarylboranes in solution.^[79]^ They again found trimesitylborane **17** to be more stable than triphenylborane **2**. To investigate further the influence of different substituents on the stability of triarylboranes, Grisdale et al. had a closer look at the influence of the *para*‐substituent in various dimesitylphenylboranes.^[80]^ To synthesize a variety of these new triarylboranes **20**, this group was the first to combine the methods previously developed by different groups. First, Grisdale and co‐workers isolated fluorodimesitylborane **18** as reported by Brown.^[76]^ This fluoroborane was then added to a lithiated species prepared from the corresponding halogenated aromatics yielding **20 a**–**e** in 40–90 %, as Wittig et al. had found lithium reagents to be suitable to react with boronhalides.^[73]^ This reaction sequence reflects the different reactivities of Grignard and organolithium reagents. Grisdale et al. also conducted one of the first systematic investigations of the photophysical properties of the new triarylboranes in various solvents, observing emission solvatochromism, suggesting the stabilization of charge transfer excited states in polar solvents.

### 1.3 Metal‐boron exchange reactions for the synthesis of triarylboranes

**Scheme 10:**
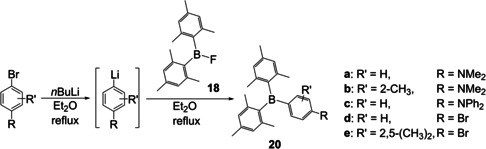
Synthetic route to symmetrically substituted triarylboranes **20 a**–**e** reported by Grisdale et al.^[80]^

To date, the most widely used method for the synthesis of triarylboranes is the procedure developed by Grisdale and co‐workers (Scheme 10)^[80]^ i.e., reaction of BF_3_ with either Grignard reagents or lithium reagents as discovered by Krause et al.^[61]^ and Wittig et al.,^[73]^ respectively. However, mercury, zinc, copper, silicon, and tin reagents have also been employed in the synthesis of triarylboranes with different reactivities, solvent compatibilities, stabilities, and accessibilities of these organometallic reagents. Furthermore, while mercury and tin reagents are not widely used currently due to their toxicities, other safety aspects may dominate the choice of organometallic reagent, depending on the organic group to be transferred.

**Scheme 11:**
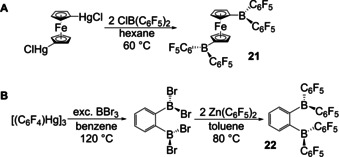
A) Hg‐B exchange reaction by Piers and co‐workers.^[83]^ B) Sequential Hg‐B and Zn–B exchange reaction by Piers, Collins, Marder and co‐workers.^[84]^

Arylmercury compounds were the first ArM reagents to be used for the synthesis of arylboranes (vide supra), but apart from a few reports on arylboronic acid syntheses,[^81^, ^82^] they have generally been replaced by Grignard or organolithium reagents. However, in 2001, Piers and co‐workers obtained the diborylated ferrocene compound **21** by reacting 1,1’‐Fc(HgCl)_2_ with ClB(C_6_F_5_)_2_ (Scheme 11 A).^[83]^ The same group made use of Hg‐B exchange to generate the diborylated compound **22**, which was then converted into a triarylborane via Zn–B exchange (Scheme 11 B).^[84]^ A year before, they reported Zn(C_6_F_5_)_2_ as a potential C_6_F_5_ transfer agent, which reacted with BCl_3_ to generate inseparable mixtures of mono‐, di‐ and triarylboranes.^[85]^

**Scheme 12:**
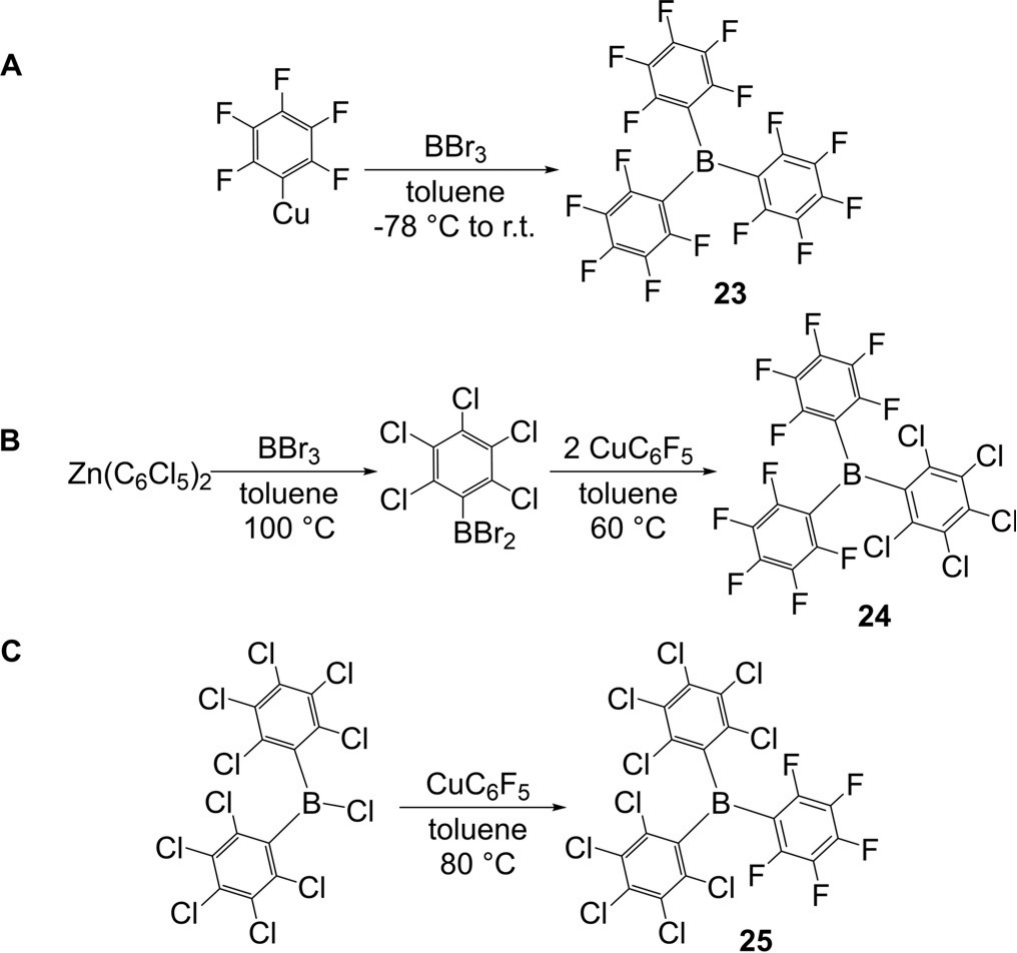
Triarylborane formation using CuC_6_F_5_ as an aryl transfer reagent by Jäkle and co‐workers and O'Hare and co‐workers, respectively.[^86^, ^87^]

In 2003, Jäkle et al. demonstrated the applicability of arylcopper reagents in Cu–B exchange reactions.^[86]^ Using mesitylcopper, a maximum of two arenes were attached even when the reaction with BX_3_ (X = Cl, Br) was heated to 100 °C, or when dichlorophenylborane **1** was used as the starting material to decrease the steric demand around the boron. Reaction of C_6_F_5_Cu with BX_3_ at room temperature gave B(C_6_F_5_)_3_ **23** irrespective of stoichiometry (Scheme 12 A). Pentafluorophenylcopper was also employed by Ashley, O'Hare and co‐workers as an aryl transfer reagent for the synthesis of triarylboranes **24** and **25** (Scheme 12 B, C) and, in one case, they made use of a Zn–B exchange to form the dibromoarylborane precursor (Scheme 12 B).^[87]^

Later, Jäkle and co‐workers demonstrated that 2,4,6‐tri‐*iso*‐propylphenylcopper (CuTip) could also be employed in Cu–B exchange reactions. Thus, CuTip was reacted with sterically unhindered bromodiarylboranes to add the third arene to the boron center,[^88^, ^89^] and these triarylborane precursors were used in the formation of organoborane macrocycles and borazine oligomers.

**Scheme 13:**
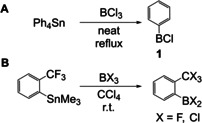
Formation of monoarylboranes via Sn–B exchange by Burch et al. and Chivers, respectively.[^90^, ^91^]

Apart from Grignard and lithium reagents, the most widely used substrates for exchange reactions with boron are organosilanes and organotin reagents. Aryltin reagents were used in Sn–B exchange reactions in the 1960s. In a first approach by Burch et al., the phenyl groups of SnPh_4_ were transferred to BCl_3_ to give compound **1**.^[90]^ Reaction of SnPh_4_ with BCl_3_ in CH_2_Cl_2_ transferred one of the four phenyl rings from tin to boron. Without the use of solvent, and under reflux conditions, all four rings were transferred (Scheme 13 A). In 1970, a more selective method was reported by Chivers, who synthesized *ortho*‐substituted monoarylboranes from the corresponding monoaryltrimethylsilanes according to Scheme 13 B.^[91]^ Halogen exchange between BCl_3_ and the *ortho*‐trifluoromethyl group was observed.

**Scheme 14:**
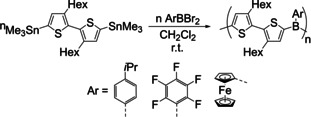
Formation of triarylborane‐containing polymers via Sn–B exchange.^[92]^

In 2005, Jäkle and co‐workers reported the synthesis of triarylborane‐containing polymers via Sn‐B exchange,^[92]^ reacting a distannylated bithiophene precursor with different dibromoarylboranes to obtain the respective polymers (Scheme 14).

Based on this procedure, many different triarylborane‐containing polymers and triarylborane model compounds were also synthesized.[^93^, ^94^]

**Scheme 15:**
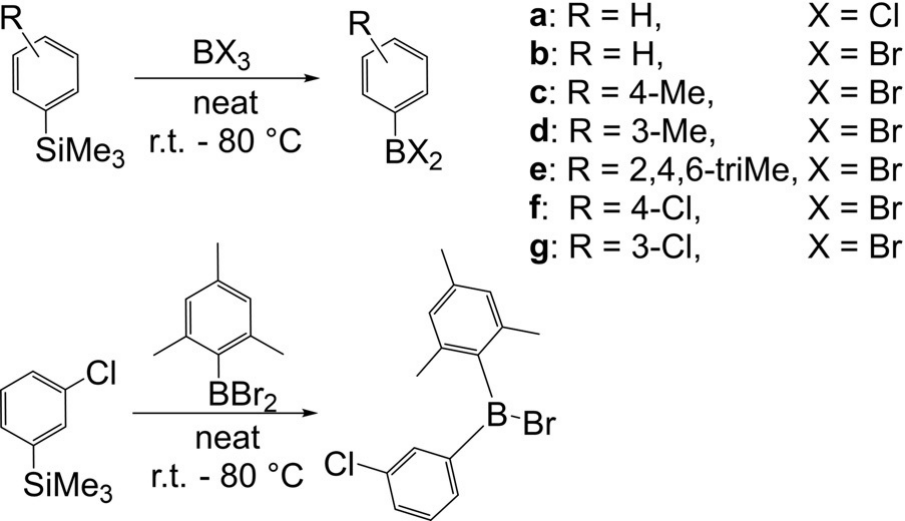
Si–B exchange reactions reported by Haubold and co‐workers.^[95]^

In 1986, Haubold and co‐workers reported the synthesis of several mono‐ and diarylboranes via Si–B exchange.^[95]^ The exchange reaction was tolerant to some functional groups, and even an unsymmetrically substituted ArAr'BBr compound was generated (Scheme 15). Starting from BBr_3_, 35 % of triphenylborane **2** was formed under harsh reaction conditions, whereas starting from BCl_3_ gave the monoarylboranes exclusively.

**Scheme 16:**
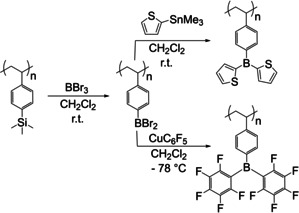
Si–B exchange reaction followed by Sn–B or Cu–B exchange for triarylborane formation by Jäkle and co‐workers.[^99^, ^100^]

Further studies as well as potential applications were reported one year later by Kaufmann et al.[^96^, ^97^] and Snieckus et al.^[98]^ Jäkle and co‐workers reported an efficient method for the introduction of a triarylborane moiety into the side chain of polystyrene.[^99^, ^100^] The first step involved Si–B exchange and, in the next step, the triarylborane was formed via Sn–B and Cu–B exchanges, respectively (Scheme 16).

**Scheme 17:**
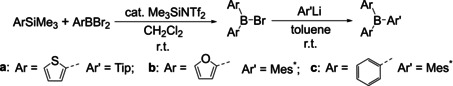
Catalyzed Si–B exchange followed by Li–B exchange for triarylborane formation.^[101]^

More recently, Helten and co‐workers improved the Si‐B exchange reaction significantly by employing a catalytic amount of Me_3_SiNTf,^[101]^ synthesizing three triarylboranes via Si–B exchange and subsequent Li–B exchange reactions (Scheme 17). Helten and co‐workers employed this method for the synthesis of triarylborane‐containing macromolecules and polymers. In each case, the third arene was attached to the boron using an aryl lithium reagent.[^102^, ^103^]

### 1.4 Potassium aryltrifluoroborates as boron sources

**Scheme 18:**
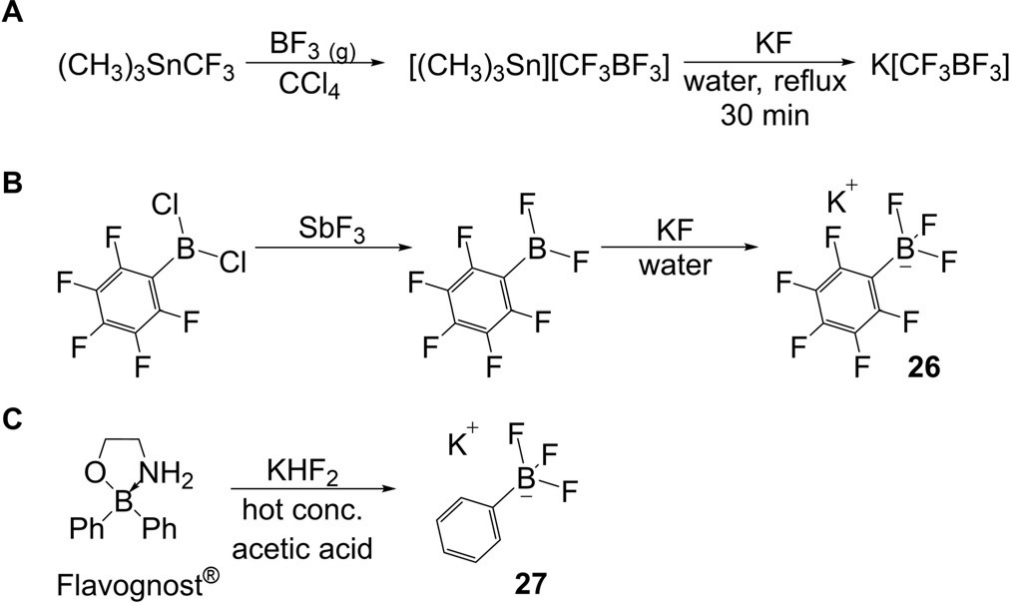
Synthetic pathways to potassium trifluoroborates by Chambers, Stafford, and Thierig, respectively.[^104^, ^105^, ^107^]

Potassium aryltrifluoroborates (ArBF_3_K salts) have been known since 1960.^[104]^ Chambers et al. reported the synthesis of potassium (trifluoromethyl)trifluoroborate from a boiling, aqueous solution of trimethyltin (trifluoromethyl)trifluoroborate and potassium fluoride (Scheme 18 A). In 1963, Stafford reported the synthesis of a potassium vinyltrifluoroborate that was isolated in a similar way to that previously described by Chambers.^[105]^ Two years later, Chambers reported the synthesis of an aromatic potassium trifluoroborate **26** which was obtained from reaction of (pentafluorophenyl)difluoroborane and potassium fluoride (Scheme 18 B).^[106]^ In 1967, Thierig and Umland reported the synthesis of potassium phenyltrifluoroborate **27** from Flavognost^®^ and potassium bifluoride (Scheme 18 C).^[107]^

**Scheme 19:**
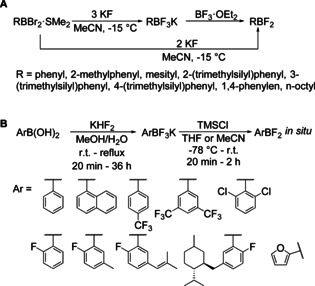
A) Conversion of aryldibromoboranes to potassium aryltrifluoroborates or aryldifluoroboranes.^[108]^ B) A convenient route to potassium aryltrifluoroborates and their activation reactions.^[109]^

About 20 years later, Kaufmann and co‐workers made use of the solubility of potassium fluoride in acetonitrile to convert RBBr_2_ compounds into their RBF_2_ analogues or the corresponding potassium trifluoroborates (Scheme 19 A).^[108]^ They also found BF_3_⋅OEt_2_ to be a suitable reagent to convert the latter salts in situ into RBF_2_ compounds.

Another way to activate potassium aryltrifluoroborates was reported by Vedejs in 1995,^[109]^ who showed that potassium aryltrifluoroborates can be activated in situ to form aryldifluoroboranes by addition of trimethylsilyl chloride (TMSCl). Furthermore, they provided a convenient route to potassium aryltrifluoroborates from the corresponding boronic acids and potassium bifluoride, KHF_2_ (Scheme 19 B).

To date, BF_3_K salts are mostly employed in reactions in which the boron motif is lost, for example, in coupling reactions.^[110]^ However, such compounds can also be used as the boron source for the syntheses of triarylboranes.

**Scheme 20:**
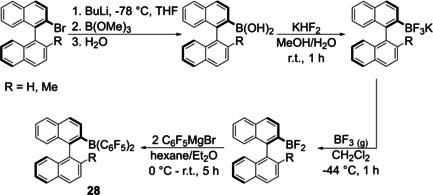
First reported synthesis of triarylboranes from potassium aryltrifluoroborates.^[27]^

In 2004, Morrison et al. were the first to synthesize triarylboranes **28** from potassium aryltrifluoroborate reagents which were activated with BF_3_ etherate and then reacted with the Grignard reagent C_6_F_5_MgBr (Scheme 20).^[27]^

**Scheme 21:**
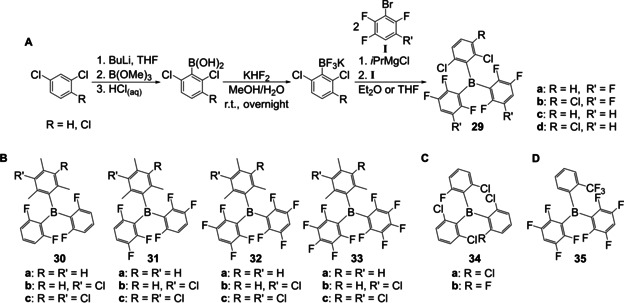
A) General reaction sequence for the synthesis of multi‐halogenated triarylboranes.^[28]^ B,^[111]^ C,^[112]^ D)^[113]^ Structures of compounds **29**–**35** synthesized according to Scheme 21 A.

**Scheme 22:**
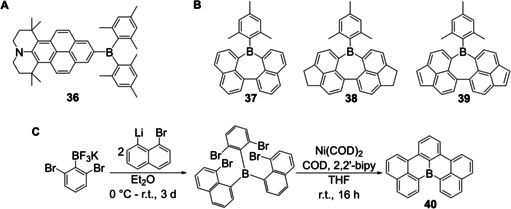
A) Structure of compound **36**.^[30]^ B) Structures of compounds **37**–**39**.^[114]^ C) Synthetic route to planarized triarylboranes.^[29]^

Since then, a few other groups reported the synthesis of triarylboranes from these bench‐stable boron precursors. Especially for applications in frustrated Lewis pairs, this approach was used for the synthesis of triarylboranes bearing aromatic systems in which multiple fluoro‐ and chloro‐substituents are desired. Soós and co‐workers[^28^, ^111^, ^112^] and Hoshimoto et al.^[113]^ synthesized triarylboranes **29**–**35** from potassium aryltrifluoroborates and Grignard reagents without prior activation of the BF_3_K salt. The Grignard reagents in these cases were each prepared from the corresponding brominated precursor in combination with the so called “Turbo‐Grignard” *iso*‐propyl magnesium chloride lithium chloride (*i*PrMgCl⋅LiCl) as summarized in Scheme 21 A. A very similar strategy, without the use of the Turbo‐Grignard, was used by Marder and co‐workers to synthesize a push‐pull system with a pyrene core **36** (Scheme 22 A).^[30]^

In contrast, Wagner and co‐workers synthesized triarylboranes as precursors to polycyclic aromatic hydrocarbons^[29]^ or quadruply annulated borepins.^[114]^ In both cases, the required triarylboranes were synthesized from potassium aryltrifluoroborates which were reacted with various aryl lithium reagents yielding compounds **37**–**40** (Scheme 22 B, C).

### 1.5 Direct dimesitylborylation

**Scheme 23:**
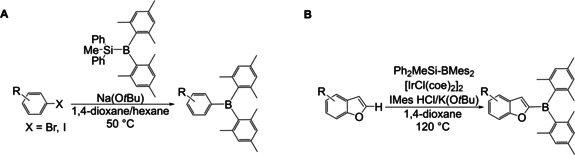
A) Dimesitylborylation of aryl halides.^[115]^ B) Dimesitylborylation of benzofuran derivatives.^[116]^

Ito and co‐workers reported the direct dimesitylborylation of various aryl halides^[115]^ by reaction of (diphenylmethylsilyl)dimesitylborane with aryl halides in the presence of a base (Scheme 23 A). The halide was replaced by boron or silicon in a ratio of ca. 9 to 1. Furthermore, the reaction was tolerant to several functional groups, and the resulting triarylboranes were isolated in moderate to good yields. In 2019, the same group reported an iridium‐catalyzed C−H dimesitylborylation of benzofuran using a silyldimesitylborane reagent (Scheme 23 B),^[116]^ preparing several derivatives and isolating the triarylboranes in moderate to good yields. Under optimized conditions, they reported the formation of the silylated side product in ca. 29 % yield.

## 2 Synthesis of Unsymmetrically Substituted Triarylboranes

Thus far, we have summarized the syntheses of symmetrically substituted BAr_3_ and BAr_2_Ar’ triarylboranes. The synthesis of unsymmetrically substituted BArAr'Ar’’ triarylboranes bearing three different aromatic rings bound to the boron center can be achieved by different routes, most of which use the same approaches used for the syntheses of symmetrically substituted triarylboranes. However, other routes employed symmetrically substituted triarylboranes as precursors.

One of the first unsymmetrically substituted triarylboranes was reported in 1971 by Grisdale et al. at Eastman Kodak.^[117]^ As mentioned above, this group investigated the photolysis of triarylboranes and tetraarylborates. During these studies, they observed an organoboron compound, formed after irradiation of potassium dimesityldiphenylborate, which contained three different aromatic systems bound to the boron center.

### 2.1 Boronic esters as boron sources

**Scheme 24:**
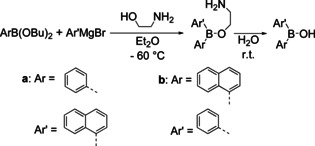
Synthesis of an unsymmetrical borinic acid by Letsinger and co‐workers.^[118]^

In 1955, Letsinger and co‐workers reported the synthesis of the first unsymmetrically substituted borinic acid starting from a boronic ester.^[118]^ They reacted phenylboronic acid butyl ester with α‐naphthylmagnesium bromide and isolated the borinic acid as its β‐aminoethyl ester, which was readily hydrolyzed to the borinic acid (Scheme 24). They also demonstrated that the synthesis works when the aryl starting materials are switched to α‐naphthylboronic acid butyl ester and phenylmagnesium bromide, respectively, but the product was not used for the synthesis of a BArAr'Ar’’ triarylborane.

**Scheme 25:**
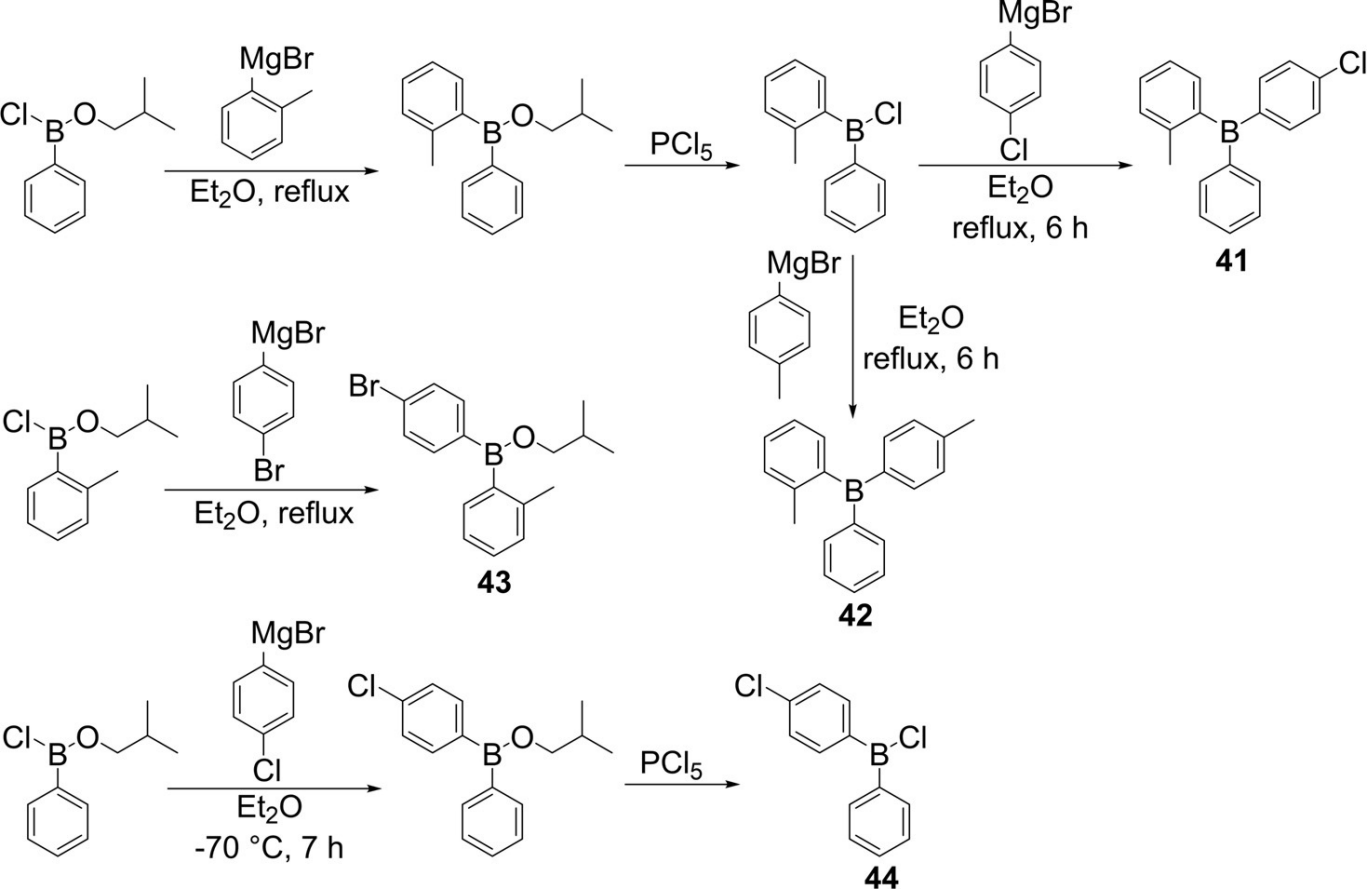
Syntheses of unsymmetrically substituted triarylboranes **41** and **42**.^[119]^

In 1958, Mikhailov et al. reported the sequential synthesis of unsymmetrically substituted triarylboranes^[119]^ (Scheme 25) starting from an *iso*‐butyl borinic ester wherein the boron atom is additionally bound to one phenyl and one chlorine atom, respectively. In the first step, the chlorine atom was substituted by an *o*‐tolyl group introduced from a Grignard reagent. In the second step, the *iso*‐butyl substituent was converted to a chloride via reaction with PCl_5_. The chlorine atom was subsequently substituted by other arenes introduced from Grignard reagents yielding two different unsymmetrically substituted triaryboranes (**41** and **42**). Mikhailov and co‐workers reported the synthesis of two other borinic acids (**43** and **44**), but their conversion to unsymmetrically substituted triarylboranes was not described.

**Scheme 26:**
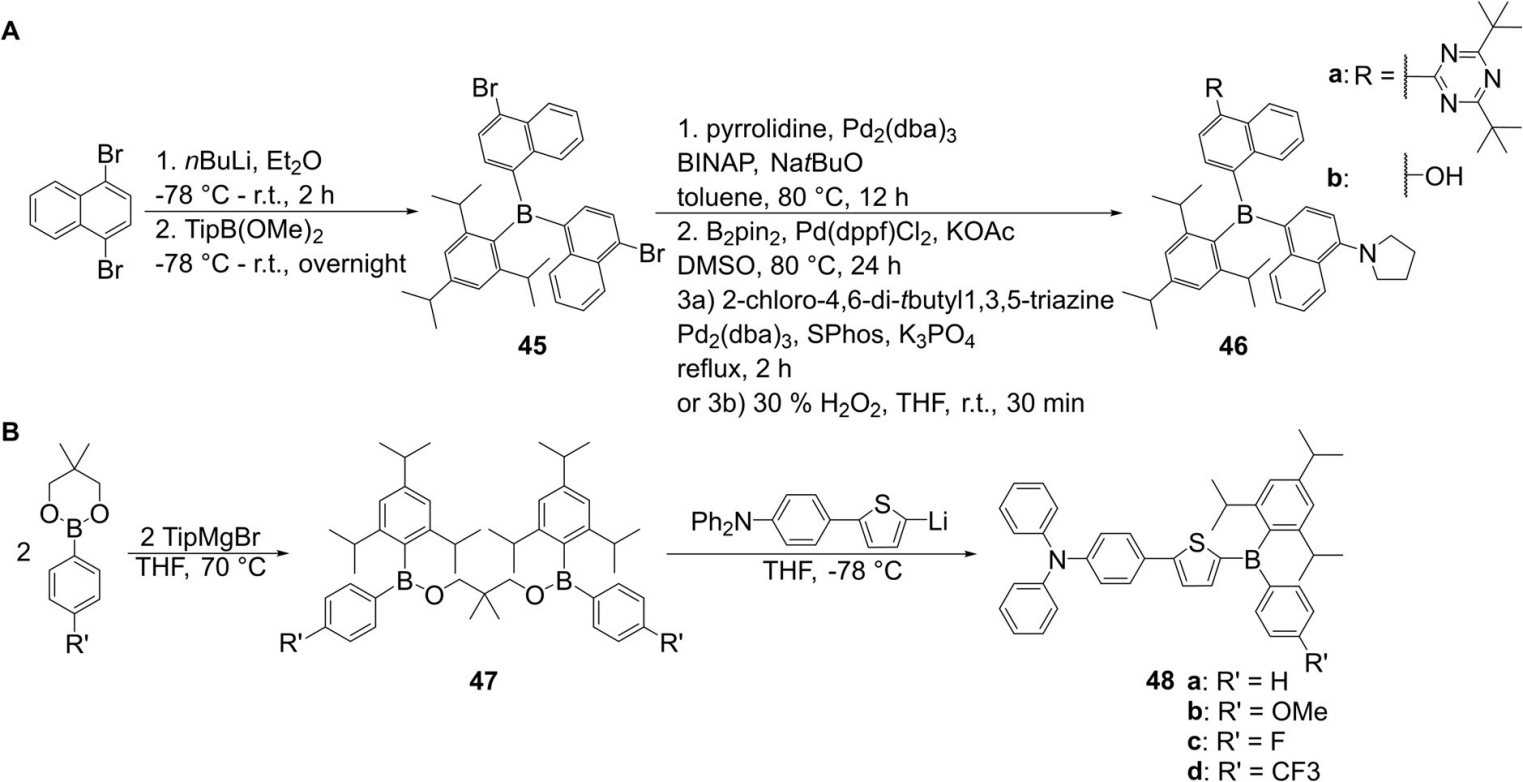
A) Synthesis of unsymmetrically substituted triarylborane **46** from a symmetrically substituted precursor.[^7^, ^120^] B) Synthesis of unsymmetrically substituted triarylboranes **48 a**–**d** from a boronic ester.^[32]^

Recently, Liu et al.[^7^, ^120^] synthesized the unsymmetrically substituted triarylboranes **46 a, b** from BAr_2_Ar’ **45**. Compound **45** was synthesized from the boronic ester TipB(OMe)_2_ (Tip = Tri‐*iso*‐propyl) which was obtained from reaction of trimethoxyborane (B(OMe)_3_) with 2,4,6‐tri‐*iso*‐propylphenyl magnesium bromide (TipMgBr).^[121]^ This boronic ester was then converted to the symmetrically substituted triarylborane **45** by reaction with a lithiated species (Scheme 26 A). Stepwise substitution of the bromides then yielded the unsymmetrically substituted triarylboranes **46 a**, **b** (Scheme 26 A).

Yamaguchi and co‐workers reported the synthesis of a series of unsymmetrically substituted triarylboranes **48 a**–**d** from a boronic ester precursor (Scheme 26 B).^[32]^ This was then converted with TipMgBr to a dimeric intermediate **47**, which was cleaved by the addition of a lithiated species yielding compounds **48 a**–**d** (Scheme 26 B). In the same paper, Yamaguchi and co‐workers reported the synthesis of a derivative of compound **48** bearing *tert*‐butyl groups instead of the *iso*‐propyl groups, but it was not possible to synthesize **48 e** according to Scheme 26 B. Therefore, they used a different approach starting from boron tribromide (vide infra, Scheme 30 B).

### 2.2 Borane dimethyl sulfide as the boron source

**Scheme 27:**
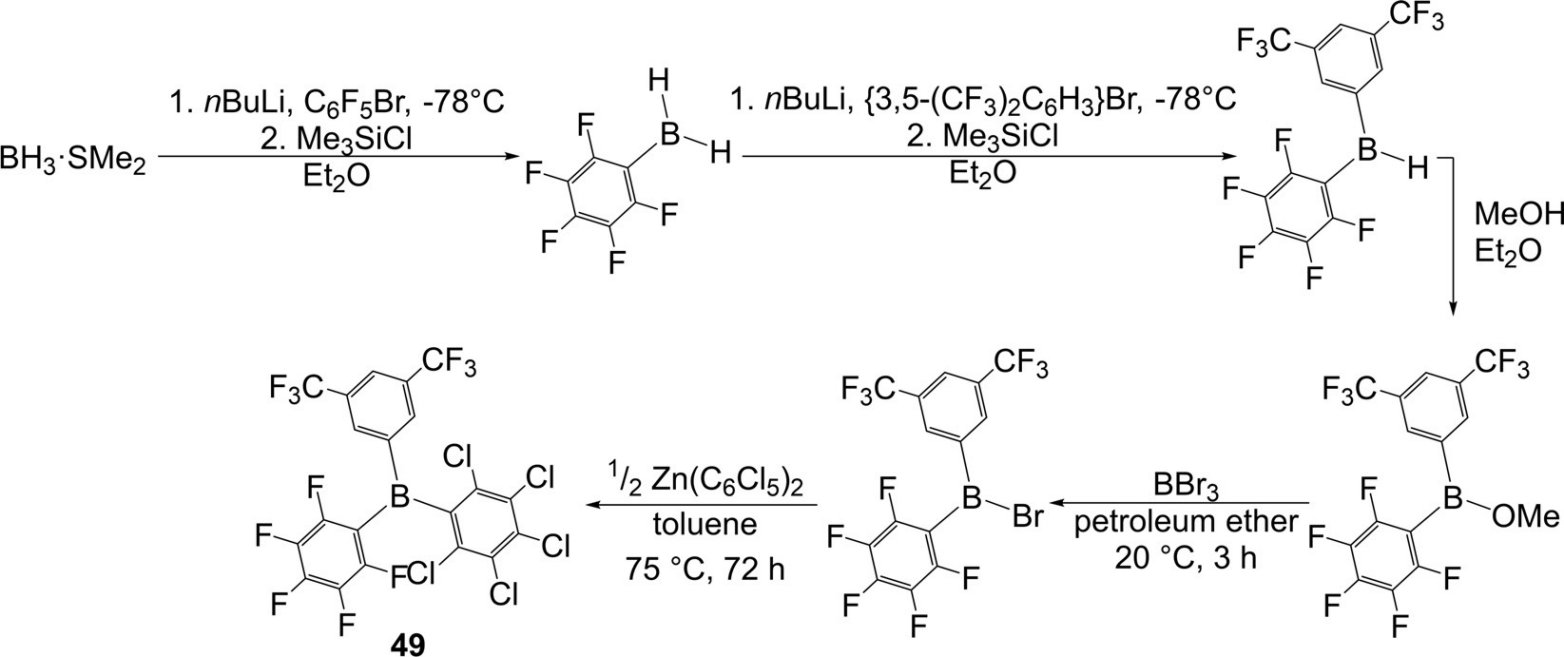
Synthesis of unsymmetrically substituted triarylborane **49** from borane dimethyl sulfide.^[122]^

In 2016, Blagg et al. reported the synthesis of the “first 1:1:1 hetero‐tri(aryl)borane”, by their own account.^[122]^ In terms of investigating the Lewis acidity of such “hetero‐tri(aryl)boranes”, they substituted the hydrogen atoms of a borane dimethyl sulfide complex stepwise with arenes (Scheme 27). The first aromatic groups were introduced using aryl lithium reagents. The resulting intermediate was converted to a borinic ester with methanol and was then activated with BBr_3_ for reaction with an organozinc reagent yielding the unsymmetrically substituted triarylborane **49**.

### 2.3 Boron trifluoride as the boron source

**Scheme 28:**
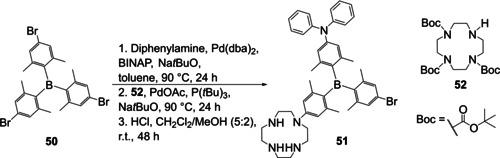
Synthesis of unsymmetrically substituted triarylborane **51** by stepwise modification of a symmetrically substituted precursor.^[123]^

Liu et al. used the symmetrically substituted compound **50**, which was prepared from BF_3_⋅OEt_2_ and the respective aryl lithium reagent, to prepare unsymmetrically substituted triarylborane **51** via sequential cross‐coupling reactions (Scheme 28).^[123]^

### 2.4 Boron tribromide as the boron source

**Scheme 29:**
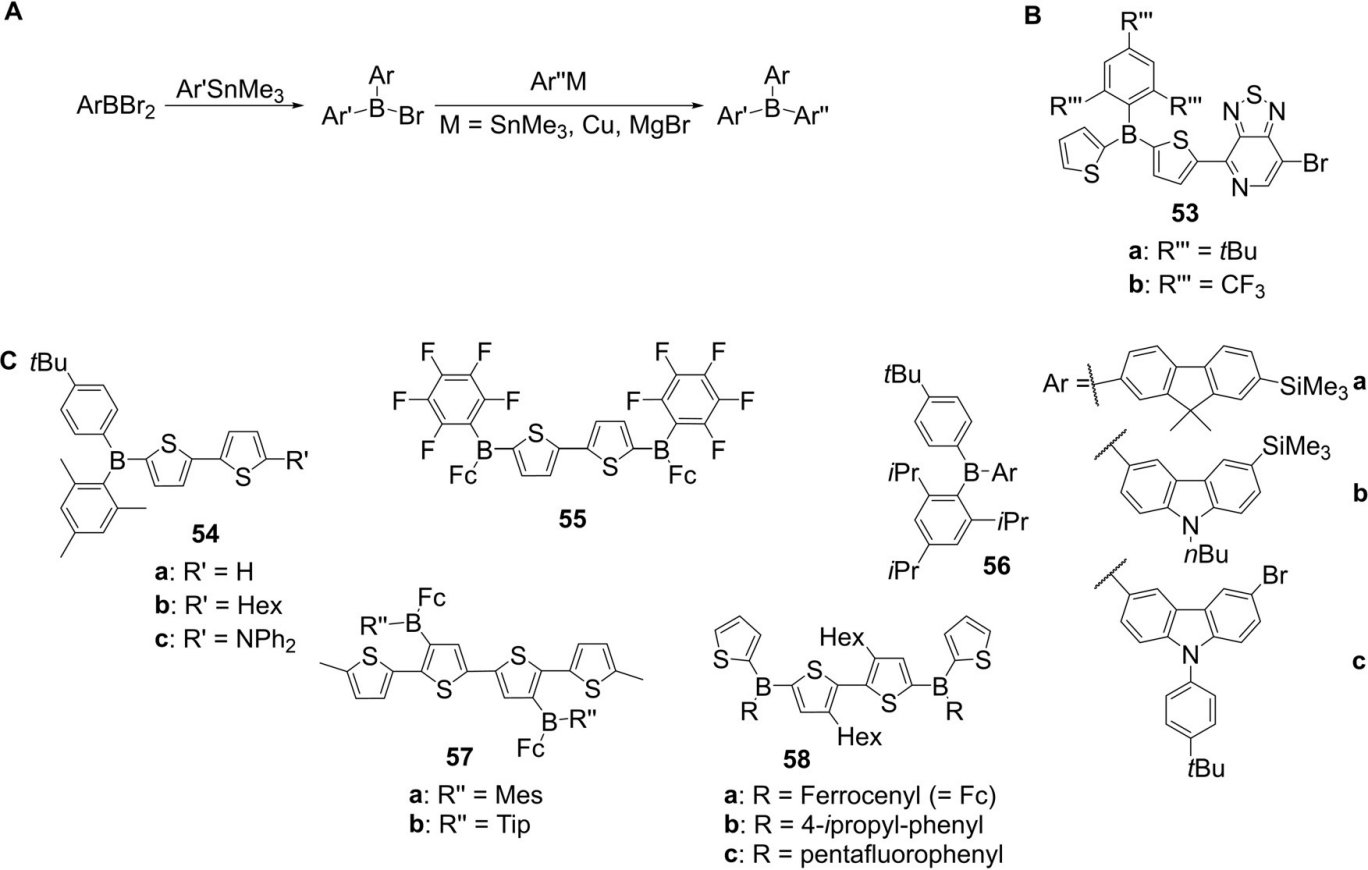
A) Synthetic route to unsymmetrically substituted triarylboranes developed by Jäkle and co‐workers.^[92]^ B) Structure of compound **53**.^[128]^ C) Compounds **54**–**58** synthesized according to Scheme 29 A.[^92^, ^124^, ^125^, ^126^, ^127^, ^128^]

In 2005, Jäkle and co‐workers reported the synthesis of different unsymmetrically substituted triarylboranes as reference compounds for their polymers.^[92]^ Both monomeric and polymeric boron‐containing systems were synthesized from aryldibromoboranes and organotin reagents (vide supra, Scheme 14; vide infra, Scheme 29). They subsequently used this strategy for similar applications with slight modifications of the synthetic procedure, the third aryl group being added via a tin,^[92]^ a copper[^124^, ^125^, ^126^] or a Grignard reagent^[127]^ (Scheme 29).

The same group then reported the synthesis of an unsymmetrically substituted triarylborane **53** (Scheme 29 B) via stochiometric Stille coupling of a symmetric precursor^[128]^ which had been obtained from boron tribromide and an excess of a tin reagent.^[129]^

**Scheme 30:**
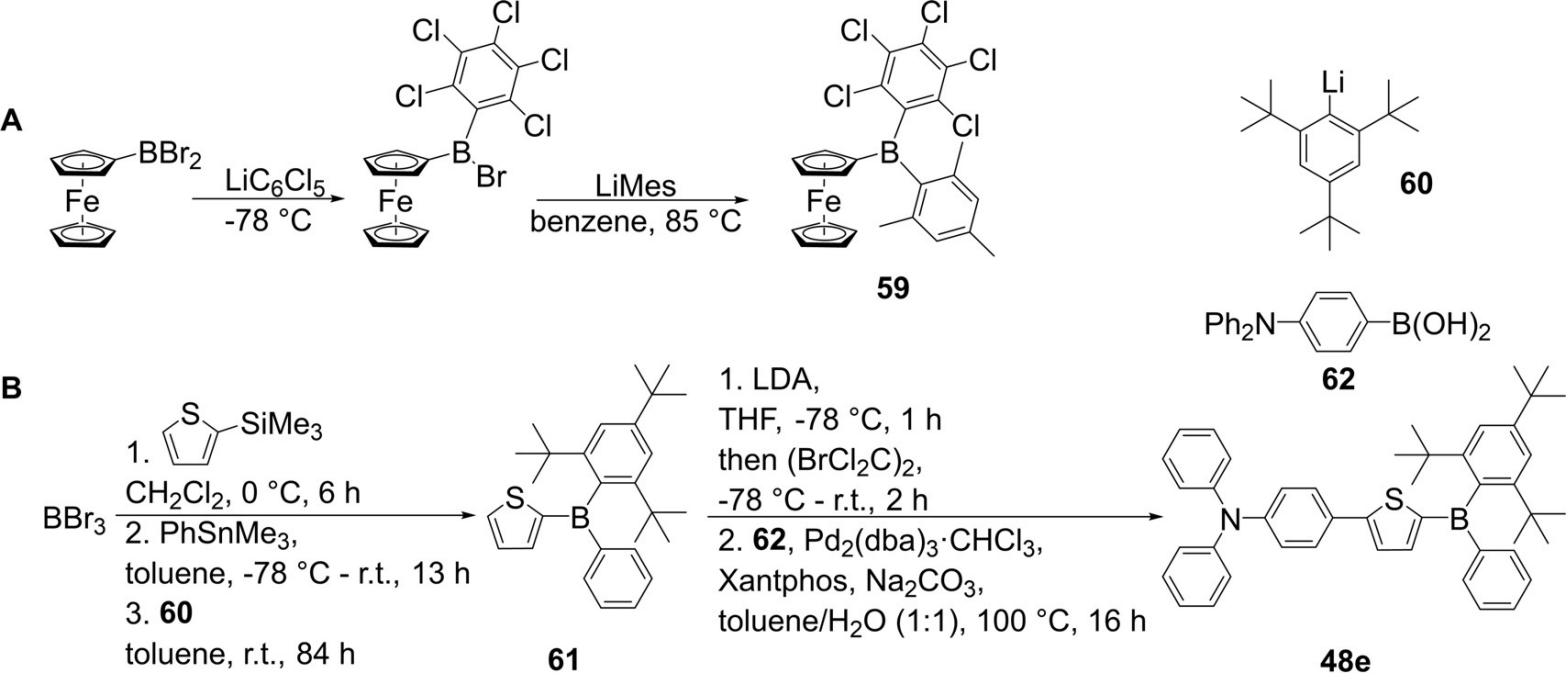
A) Synthesis of unsymmetrically substituted triarylborane **59** by Kelly et al.^[130]^ B) Synthesis of sterically more demanding, unsymmetrically substituted triarylborane **48 e** by Yamaguchi and co‐workers.^[32]^

In 2014, Kelly et al. reported the synthesis of a ferrocene‐containing triarylborane bearing three different aromatic systems by stepwise reaction of dibromoferrocenylborane with two different aryl lithium reagents (Scheme 30 A).^[130]^

As shown in Scheme 26 B, Yamaguchi and co‐workers reported a route to unsymmetrically substituted triarylboranes from boronic esters,^[32]^ but for **48 e**, the route was not successful as the incorporated arene was sterically too demanding. Therefore, they used a route established by Jäkle and co‐workers: after a boron–silicon exchange at the thiophene, the ArBBr_2_ system was reacted with a tin reagent followed by 2,4,6‐tri‐*tert*‐butylphenyllithium to give **48 e** (Scheme 30 B).

## Summary and Outlook

Over the years, the synthetic approaches to triarylboranes presented herein has led to the generation of countless compounds containing triarylborane motifs. Initially, examination of their properties was limited to their reactivity with other metals or as Lewis acids. Some of the early reaction sequences, such as those developed by Krause et al. and Grisdale et al., are still used. Today, the applications of these compounds are no longer limited to their reactivity. The photophysical and electronic properties of triarylboranes and compounds containing this structural motif remain under increasingly active investigation as such properties lead to numerous applications, for example, in OLEDS,^[2]^ optoelectronics,[^1^, ^3^, ^23^] sensors for anions[^4^, ^5^, ^6^] or small molecules,[^7^, ^8^] as catalysts, for example, for hydrogenation or amination of carbonyls,[^28^, ^111^, ^112^] or bioimaging agents.[^9^, ^10^, ^12^, ^14^, ^15^] With the further exploration of more general routes to unsymmetrically substituted triarylboranes, the applicability of these compounds can be expected to continue to increase as this structural motif provides the possibility for fine tuning of the photophysical and electronical properties of the resulting small molecules and, therefore, also of potential macromolecules and polymers.

## Conflict of interest

The authors declare no conflict of interest.

## Biographical Information

*Sarina M. Berger studied chemistry at the Julius‐Maximilians‐University of Würzburg and completed her Bachelor's thesis in Prof. Dr. Frank Würthner's group at the Institute of Organic Chemistry in 2016. She then joined Prof. Dr. Todd B. Marder's group at the Institute of Inorganic Chemistry where she completed her Master's thesis in 2018*, *and is currently carrying out her PhD research on the synthesis and opto‐electronical properties of selectively charged triarylborane chromophores*.

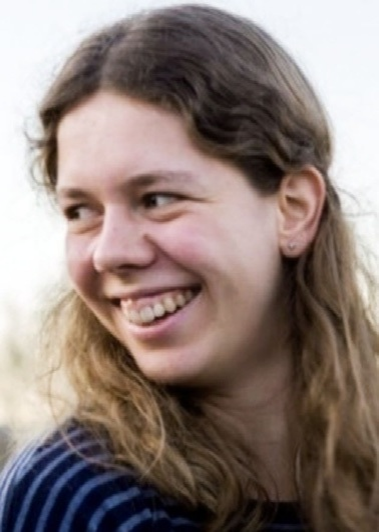

## Biographical Information

*Matthias Ferger studied chemistry at the Julius‐Maximilians‐University of Würzburg. He completed his Bachelor's thesis in the group of Prof. Dr. Udo Radius on anionic sp^2^–sp* ^*3*^ *adducts of diboron(4) compounds and received his BSc degree in 2014. He then joined Prof. Dr. Todd B. Marder's group*, *completed his Master's thesis in 2016 and is currently carrying out his PhD research on the development of methodology for the synthesis of triarylboranes*, *and on the synthesis and photophysical properties of triarylborane chromophores for DNA and RNA sensing*.

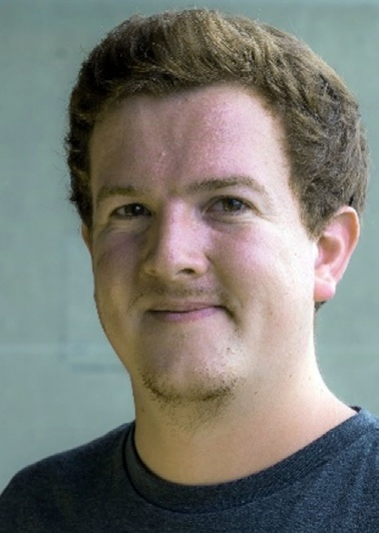

## Biographical Information

*Todd Marder obtained his BSc from M.I.T. and his PhD from UCLA (Regents Intern Fellow). He was a postdoc at the University of Bristol (UK)*, *and a Visiting Research Scientist at DuPont Central Research before joining the faculty at the University of Waterloo*, *Canada. He moved to the University of Durham (UK) in 1997 as Chair of Inorganic Chemistry and then to the University of Würzburg*, *Germany in 2012*, *also as Chair of Inorganic Chemistry. Honors include: the Rutherford Memorial Medal for Chemistry (Royal Society of Canada)*, *RSC (UK) Awards in Main Group Element Chemistry and in Organometallic Chemistry*, *JSPS Fellowship*, *Humboldt Research Award*, *Royal Society Wolfson Research Merit Award*, *elected member of the Bavarian Academy of Sciences*, *fellow of the Royal Society of Chemistry (FRSC)*, *the American Association for the Advancement of Science (AAAS)*, *and the European Academy of Science (EurASc)*, *Visiting/Honorary/Distinguished/Guest Professorships in the UK*, *France*, *Hong Kong*, *mainland China*, *Japan*, *India and the Craig Lectureship in Australia*.